# Working memory predictors of mathematics across the middle primary school years

**DOI:** 10.1111/bjep.12339

**Published:** 2020-01-30

**Authors:** Katie Allen, David Giofrè, Steve Higgins, John Adams

**Affiliations:** ^1^ School of Education University of Durham UK; ^2^ Dipartimento di Scienze della Formazione (DISFOR) University of Genoa Italy; ^3^ School of Education University of Durham UK; ^4^ Department of Psychology University of Durham UK

**Keywords:** verbal, visuospatial, mathematics, children

## Abstract

**Background:**

Work surrounding the relationship between visuospatial working memory (WM) and mathematics performance is gaining significant traction as a result of a focus on improving academic attainment.

**Aims:**

This study examined the relative contributions of verbal and visuospatial simple and complex WM measures to mathematics in primary school children aged 6–10 years.

**Sample:**

A sample of 111 children in years 2–5 were assessed (*M*_age_ = 100.06 months, *SD* = 14.47).

**Method:**

Children were tested individually on all memory measures, followed by a separate mathematics testing session as a class group in the same assessment wave.

**Results and Conclusions:**

Results revealed an age‐dependent relationship, with a move towards visuospatial influence in older children. Further analyses demonstrated that backward word span and backward matrices contributed unique portions of variance of mathematics, regardless of the regression model specified. We discuss possible explanations for our preliminary findings in relation to the existing literature alongside their implications for educators and further research.

There is an increasing wealth of literature on the relationship between working memory (WM) and academic attainment in school‐aged children. WM can be operationally defined as the capacity to temporarily store and manipulate information, necessary for the completion of complex tasks (Baddeley, 1992). The model of WM proposed by Baddeley and Hitch (1974) has been developed since its conception to include two slave systems, the visuospatial sketchpad and the phonological loop, responsible for the storage and manipulation of visual and verbal information, respectively (Baddeley, 2000). The visuospatial sketchpad, therefore, supports visuospatial WM, while the phonological loop supports verbal WM. This WM model continues to be robust to methodological advances and research findings, and has repeatedly been used in studies conducted with typically developing children (Alloway, Gathercole, & Pickering, 2006; Giofrè, Borella, & Mammarella, 2017; Giofrè, Mammarella, & Cornoldi, 2013).

Several authors suggest that WM is differentially related to tasks depending on their content, for example, to specific areas of mathematics (Peng, Namkung, Barnes, & Sun, 2016). In particular, numeric verbal WM seems to be more closely related to number‐based mathematical tasks (as in Raghubar, Barnes & Hecht, 2010), while visuospatial WM shows a stronger relationship with tasks with a clearer visuospatial element, for example geometry (Giofrè, Mammarella, Ronconi, & Cornoldi, 2013). Allen and Giofrè (2019) demonstrated results of this nature in 7‐ to 8‐year‐old children, suggesting one influencing factor on the extent of the influence of WM on mathematical performance lies in the WM tasks administered. Similar findings indicating the differential influence of WM components on mathematics can be found in Andersson and Lyxell (2007); Nosworthy, Bugden, Archibald, Evans, and Ansari (2013); Holmes and Adams (2006); and Holmes, Adams, and Hamilton (2008).

With regard to mathematics as a whole, results appear largely mixed, seemingly dependent on the measure of WM adopted. WM tasks can be divided into those that measure simple span (whereby participants are required to recall a list of target words/letters/digits/shapes immediately after presentation; Unsworth & Engle, 2007), complex span (whereby participants complete an unrelated processing task alongside the recall task; Unsworth & Engle, 2007), and dual tasks (tasks requiring the active manipulation of the presented stimuli before recall of any kind; McDowell, Whyte, & D’Esposito, 1997). Simple measures of span (sometimes referred to as short‐term memory tasks) do not require an extensive manipulation of the stimuli, while the so called complex span (sometimes referred to as WM tasks) requires some sort of manipulation of the stimulus and generally higher levels of cognitive control (see Engle, 2010 for more information about this distinction). On occasion, those measuring only simple span are considered to be representative of short‐term memory processes only (as in Kail & Hall, 2001); however, they are often included in WM batteries to develop a complete understanding of an individual’s memory capacity, particularly when working with young children. Alternative formulations of WM do not postulate a clear distinction between simple (i.e., short‐term memory) and complex (i.e., WM) tasks, but advance the idea that different tasks can be differentiated on a sort of continuum between simple and complex tasks (see Cornoldi & Vecchi, 2003; and Cornoldi & Giofrè, 2014 for a review). It is also noteworthy that very young children might present with some difficulties in dealing with complex tasks; hence, simple span tasks could probably provide an insight into their ability to complete tasks of this nature, with fewer task demands.

A recent systematic review by Peng, Namkung, Barnes, and Sun (2016) found a significant positive relationship between WM and mathematics, however, interestingly, no differences between the contributions of WM components to mathematics. It is important here to consider that the study compared verbal, numeric, and visuospatial WM tasks only, using a stringent definition of WM tasks as only complex span or dual tasks, which are supposed to require more attentional resources (or cognitive load) as compared to simple memory tasks (Engle, Tuholski, Laughlin, & Conway, 1999; Kane *et al.*, 2004). Taking a longitudinal approach is valuable for showing the stability of the existence of a relationship between WM and mathematics (as suggested by studies proposing a developmental shift during childhood, e.g., De Smedt *et al.*, 2009; Van de Weijer‐Bergsma, Kroesbergen & Van Luit, 2015); however, the influence of simple tasks was neglected, which may be especially important for understanding the relationship in younger children (as seen in Holmes, Adams & Hamilton, 2008).

Allen, Higgins and Adams (2019) addressed this issue with regard to visuospatial WM, similarly identifying a positive relationship between WM and mathematics when considering school‐aged children. This paper further elaborates on the important role of age in the relationship between WM and mathematics (e.g., Li & Geary, 2013; Soltanlou, Pixner & Nuerk, 2015; Van de Weijer‐Bergsma *et al.*, 2015), highlighting the cumulative nature of knowledge. Hence, mastery is sought in individual aspects of mathematics, rather than in mathematics as a whole. Further, it follows that there is evidence of a declarative shift in strategy use which may influence the components of WM accessed by mathematics questions (see Schneider, 2008 for a review of this). As such, the age of the participants will be crucial to the expected extent of involvement of each component as the pattern of involvement of WM components in mathematics varies as a function of age (Friso‐van den Bos, van der Ven, Kroesbergen & van Luit, 2013).

Taking a more holistic approach to the types of WM tasks used, Friso‐van den Bos, van der Ven, Kroesbergen, and van Luit (2013) conducted a further meta‐analysis identifying an association between WM and mathematics in 4‐ to 12‐year‐olds. In doing so, they identified an influence of age on the component of WM with the strongest influence; that is, visuospatial WM tasks were more highly correlated in younger children, with verbal WM becoming more influential as children grew older. Similarly, visuospatial WM was found to be the dominant deficit in developmental dyscalculia (Mammarella, Caviola, Giofrè, & Szűcs, 2018; Szűcs, Devine, Soltesz, Nobes, & Gabriel, 2013). Likewise, a study by McKenzie, Bull, and Gray (2003) found comparable results, showing that visuospatial WM is more strongly related to whole‐number calculations in younger children, while visuospatial and verbal WM was related to calculations in older children. Conversely, as previously mentioned, one important influence on the extent of the involvement of WM tasks may be the individual task demands as the demands of more complex WM tasks may be quite difficult for younger children. Sweller (1994) suggested that the extent to which WM components contribute may be a result of the cognitive load of each task, with multistep and word problems demanding more WM resources due to the need for more placeholding and knowledge integration. There is a clear gap in the literature here in exploring the link between task complexity, the age of the children assessed, and the predictive value of such tasks for mathematics performance.

This paper aimed to address the gaps in the literature identified above by investigating which components of WM are more influential in mathematics performance at different ages across the primary school years. The cognitive control required by each individual task has been manipulated. We used simple tasks, that is, forward span, which required a lower level of attentional control, backward span, which additionally requires children to recall the information in backward order, and dual tasks, which requires children to perform two tasks at the same time and is thought to require higher levels of attentional control. In fact, some WM models distinguish between a horizontal continuum, for example, differentiating between the verbal and visuospatial modalities, and a vertical continuum, in which tasks are differentiated based on different levels of attentional control required (see Cornoldi & Vecchi, 2000, 2003). The use of tasks tapping different levels of attentional control and targeting the visuospatial and verbal components was necessary in order to highlight the crucial relationships with mathematics over development. Based on previous work in this area (e.g., Allen *et al.*, 2019; Holmes & Adams, 2006), we would expect to see a relatively stable influence of visuospatial WM, with a shift in the strength of the relationship with verbal WM. This paper will combine both simple and complex tasks that access the verbal and visuospatial components of WM in order to provide the basis for developing a more thorough understanding of the influence of such measures on mathematical performance in children aged 6–10 years.

## Method

### Participants

The sample consisted of 111 6‐ to 10‐year‐old children. All children completed both phases of the administration within the same assessment wave; hence, the final sample was of 28 Year 2 (6–7 years), 26 Year 3 (7–8 years), 30 Year 4 (8–9 years), and 27 Year 5 (9–10 years) children (61 male and 50 female, *M*_age_ = 100.06 months, *SD* = 14.47). An opportunity sample of the four year groups from one primary school was used, using opt‐out parental consent to reduce bias in the sample (Krousel‐Wood *et al.*, 2006). The study was approved by the School of Education Ethics Committee at the University of Durham. Parental consent was assumed if no opt‐out slip was received. Children with special educational needs, intellectual disabilities, or neurological and genetic disorders were not included in the study.

### Design and procedure

All children were tested individually in a quiet area of their school. The six working memory measures were administered in a randomized order, using counterbalancing to reduce the effects of fatigue and practice. A correlational design was adopted to explore the relationships between visuospatial working memory and maths performance. All working memory measures were administered in a computerized format, using E‐Prime. Two trials of each span length were used, with children completing the whole test to provide a fully saturated measure of their WM capacity. The mathematics test was presented in paper and pencil format. Children could ask for a question to be read aloud in order to not place children of lower reading ability at a disadvantage. Partial credit score was used for all WM tasks (as in Giofrè & Mammarella, 2014) whereby participants are credited for all correct responses made in the correct serial position irrespective of whether the full response list was recalled accurately. This measure provides a fully saturated picture of an individual’s WM capacity and allows us to take into account the information from partially accurate lists. The partial credit score is more reliable and accurate as compared to traditional scoring methods, such as absolute credit score (Giofrè & Mammarella, 2014; Unsworth & Engle, 2007).

### Measures

The WM measures used in this paper demonstrated very good psychometric properties and were previously used in other studies with similar populations to the current study (e.g., Giofrè, Borella & Mammarella, 2017).

#### Verbal WM

**Figure 1:**
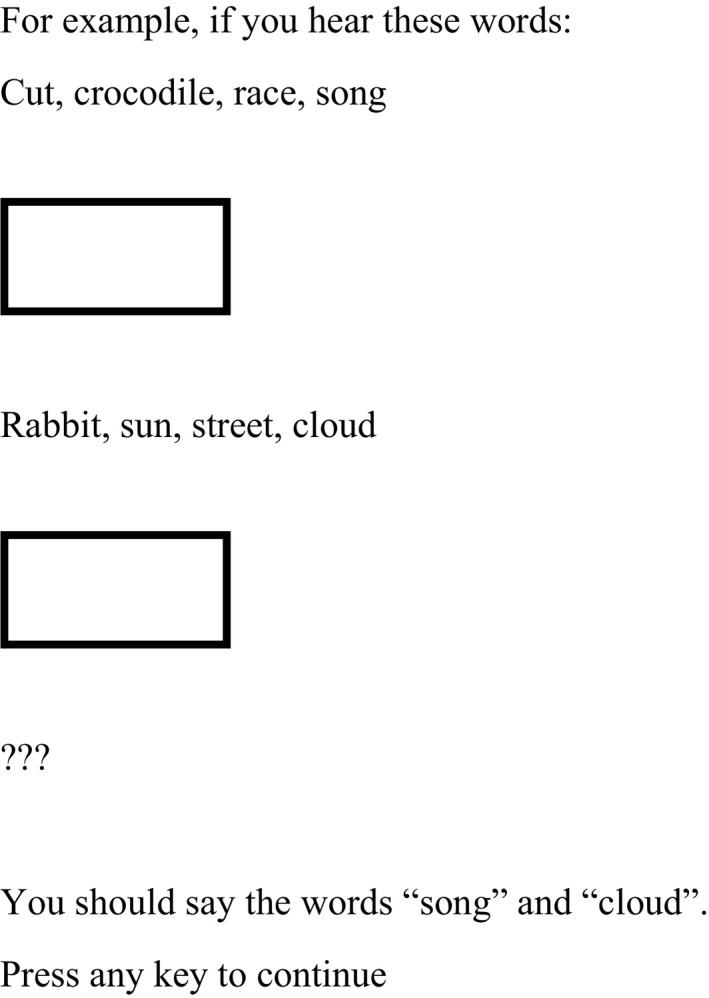
Instructions for the verbal dual task.

Three measures of verbal WM were taken: forward word span, backward word span, and a verbal dual task. Forward and backward word span tasks required children to repeat the list of words they had heard in either forward or backward order, respectively (Cronbach’s alpha .71 and .83, respectively). The dual task required children to listen to a number of word lists, all of length 4. Children were required to press the spacebar when they heard the name of an animal, as well as retaining the final word in each list (see Figure 1 for an example). None of the word lists used contained mathematical and or geometrical words, for example, rectangle or multiplication. Once they had heard all of the lists for that trial, children were asked to recall the final word from each list in the correct order (alpha = .83). All tasks presented words at a rate of one word every 2 s.

#### Visuospatial working memory

Three measures of visuospatial WM were taken: forward matrices, backward matrices, and a visuospatial dual task, using 4 × 4 grids. Forward (alpha = .72) and backward (alpha = .87) matrices required children to repeat the sequence of black squares they had seen in either forward or backward order, respectively. The dual task presented a series of grids with a number of squares coloured grey. In each grid, children saw three black dots one after the other. Children were required to press the spacebar if they saw a dot in a grey square, as well as remembering the position of the last (3rd) dot in each grid. Once they had seen all of the grids for that trial, children were asked to recall the positions of the last dots in the correct order (alpha = .82). All tasks presented stimuli at a rate of one dot/square per 2 s.

#### Mathematics

##### HeadStart Primary Mathematics.

The Head Start Primary Mathematics test is a standardized measure of mathematics, providing a year group‐specific measure of mathematical performance, in line with the objectives of the National Curriculum. Children are required to develop an understanding of number (e.g., Fill in the answer boxes. (1) 2 twos are __? (2) 11 twos are __?), measurement (e.g. A bag of apples should weigh 22 kg. One bag weighs 23.5 kg, another weighs 24 kg. How much are the bags overweight altogether?), geometry (e.g. Mrs Pott’s garden lawn is rectangular. The lawn measures 8 m by 9 m. What is the perimeter of the lawn?), and statistics (e.g., Look at the bar chart below. How many fewer people like rabbits than hamsters?) according to the National Curriculum in the United Kingdom. The number of questions addressing each of these topic areas was equal across tests. As such, it provides a comprehensive profile of how children perform when faced with questions relating to different aspects of maths. Additionally, each mathematics test is designed to be of equal difficulty, relative to the National Curriculum expectations of each year group. Children were read the instructions for the Head Start Primary Mathematics test before beginning and were allowed a maximum of 1 hr to complete the test. Each test contained 25 questions; thus, 60 min provided sufficient time for completion. The instructions given included clarification of where to write their answers, explanation that they must follow the individual instructions given for each question (e.g., use a mental/written method), and that questions may be read to them should they wish. However, no further explanation of the question, or what was required, was given. Typical classroom test conditions were adopted throughout.

### Data analytic plan

All analyses were performed using R (R Core Team, 2018). The package ‘psych’ was used to perform regressions (Revelle, 2017) and the package ‘VennDiagram’ for producing Venn diagrams (Chen, 2018). To obtain a more precise picture of the proportion of unique and shared variance among the variables, we utilized variance partitioning methods, which have been successfully used in similar studies (Giofrè, Mammarella, & Cornoldi, 2014; Unsworth & Engle, 2006). Variance partitioning, also known as commonality analysis, attempts to partition the overall *R* ^2^ of a particular criterion variable into portions that are shared and unique to a set of independent predictor variables (Pedhazur, 1997; Unsworth & Engle, 2006).

## Results

### Preliminary analyses

Descriptive statistics revealed all skewness and kurtosis values were within the bounds of +/− 1; hence, parametric tests were used throughout. Correlations (covarying for age) and descriptive statistics are presented in Table 1. We also performed the analyses for each year group. We performed a series of correlations between age in months and each WM task, and these were not statistically significant.

**Table 1:** Correlation, means, and standard deviations for each measure

	1	2	3	4	5	6	7
1. Forward word span	1						
2. Backward word span	.514**	1					
3. Verbal dual task	.533**	.347**	1				
4. Forward matrices	.443**	.391**	.376**	1			
5. Backward matrices	.426**	.453**	.330**	.569**	1		
6. Visuospatial dual task	.341**	.282**	.427**	.491**	.437**	1	
7. Mathematics	.439** ^,^ †	.613** ^,^ †	.282** ^,^ †	.405** ^,^ †	.533** ^,^ †	.375** ^,^ †	1
*M*	24.1	26.48	13.95	35.32	26.77	13.26	97.61
*SD*	7.48	6.22	7.12	9.18	12.7	7.77	13.89

Note

False discovery rate (FDR; Benjamini & Hochberg, 1995) correction of the *p*‐values (implemented using the *p*.adjust function in R) was applied across the six bivariate associations of interest, that is, between mathematics and each individual WM task.

** *p* < .01, one tail

^†^ *p* < .05, one tail, FDR correction.

### Analyses on the overall sample

We performed a series of regressions to understand the specific contribution of our predictors to mathematics for the overall group without distinguishing between different age groups.

In the first regression, verbal WM tasks (forward word span, backward word span, and a verbal dual task) were predicting mathematics. This model was statistically significant, *F*(3, 107) = 23.52, *p* < .001, *R* ^2^ = .40. In this model, backward word span, *β* = .53, 95% CI [0.35, 0.70], was predicting a significant portion of the variance of mathematics, while forward word span, *β* = .16, 95% CI [−0.03, 0.35], and verbal dual task, *β* = .01, 95% CI [−0.16, 0.19], were not predicting significant portions of the variance of mathematics.

We also performed a similar regression analysis in which visuospatial WM tasks (i.e., forward matrices, backward matrices, and a visuospatial dual task) were predicting mathematics. This model was statistically significant, *F*(3, 107) = 16.39, *p* < .001, *R* ^2^ = .31. In this model, backward matrices, *β* = .41, 95% CI [0.22, 0.61], were predicting a significant portion of the variance of mathematics, while forward matrices, *β* = .10, 95% CI [−0.11, 0.30], and visuospatial dual task, *β* = .15, 95% CI [−0.04, 0.33], were not predicting significant portions of the variance of mathematics.

In a final regression, verbal and visuospatial tasks were entered simultaneously as predictors of mathematics. This model was statistically significant, *F*(6, 104) = 15.81, *p* < .001, *R* ^2^ = .48. In this model, backward word span, *β* = .43, 95% CI [0.26, 0.60], and backward matrices, *β* = .26, 95% CI [0.07, 0.44], predicted significant portions of the variance of mathematics, while the other predictors were not statistically significant (*β*s < .13, *p*s > .05).

**Figure 2:**
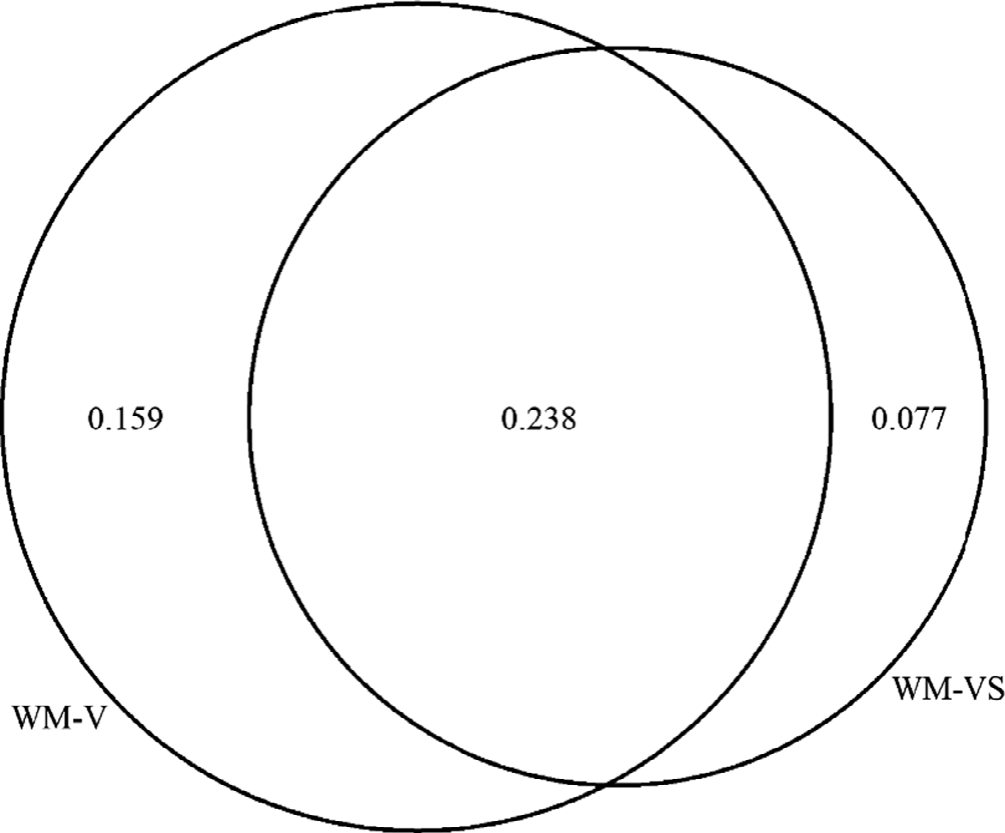
Variance decomposition. WM‐V = verbal working memory, WM‐VS = visuospatial WM.

In order to partition the variance, a series of regression analyses was carried out to obtain *R* ^2^ values from different combinations of the predictor variables (see Table 2). The results showed that a large portion of the variance was shared (Figure 2). However, both verbal and visuospatial tasks were also predicting portions of unique variance. Variance inflation (VIF) in each individual regression, presented in Table 2, was generally low, that is, lower than 2.

**Table 2:** *R* ^2^ values for regression analyses predicting mathematics for various predictor variables

Predictor variables	*R* ^2^	*F*
Visuospatial WM	.31	16.39
Verbal WM	.40	23.52
Verbal WM and visuospatial WM	.48	15.81

Note

All *R* ^2^ values are significant at *p* < .001.

WM = working memory.

### Analyses per age group

The data were broken down by year group before performing correlational analyses to investigate the strength of the respective relationships between mathematics and each WM measure, depending on the age of the children (Table 3, see also the Figures S1–S4). Results showed the strongest relationships between mathematics and verbal span backwards in Year 2, backward matrices and verbal span backwards in Year 3, forward matrices and verbal span backwards in Year 4, and backward matrices and visuospatial dual task in Year 5 (Table 3).

**Table 3:** Correlations, means, and standard deviations for each measure, distinguished by year

	1	2	3	4	5	6	7
Year 2							
1. Forward word span							
2. Backward word span	.346*						
3. Verbal dual task	.585**	.296					
4. Forward matrices	.124	.068	.189				
5. Backward matrices	.325*	.308	.168	.479**			
6. Visuospatial dual task	.470**	.047	.422*	.096	.235		
7. Mathematics	.274	.738** ^,^ †	.225	.145	.445** ^,^ †	.197	
*M*	19.32	22.00	11.39	28.54	19.00	11.54	93.68
*SD*	7.53	5.00	7.56	7.63	11.07	5.66	9.85
Year 3							
1. Forward word span							
2. Backward word span	.488**						
3. Verbal dual task	.621**	.245					
4. Forward matrices	.441*	.405*	.364*				
5. Backward matrices	.618**	.514**	.341*	.694**			
6. Visuospatial dual task	.417*	.320	.335*	.524**	.589**		
7. Mathematics	.559** ^,^ †	.718** ^,^ †	.277	.409* ^,^ †	.682** ^,^ †	.299	
*M*	24.96	27.69	13.23	36.31	30.38	10.15	99.85
*SD*	7.15	6.23	6.86	9.24	12.01	8.09	18.36
Year 4							
1. Forward word span							
2. Backward word span	.364*						
3. Verbal dual task	.436**	.330*					
4. Forward matrices	.417*	.353*	.411*				
5. Backward matrices	.119	.055	.306*	.417*			
6. Visuospatial dual task	.010	.203	.203	.500**	.313*		
7. Mathematics	.350* ^,^ †	.614** ^,^ †	.333* ^,^ †	.625** ^,^ †	.428** ^,^ †	.535** ^,^ †	
*M*	25.67	27.97	13.47	38.17	27.90	14.87	99.67
*SD*	5.71	5.73	5.54	6.86	12.02	7.87	12.24
Year 5							
1. Forward word span							
2. Backward word span	.495**						
3. Verbal dual task	.357*	.321					
4. Forward matrices	.364*	.169	.357*				
5. Backward matrices	.305	.560**	.329*	.424*			
6. Visuospatial dual task	.371*	.372*	.608**	.670**	.615**		
7. Mathematics	.458** ^,^ †	.422* ^,^ †	.348* ^,^ †	.335* ^,^ †	.500** ^,^ †	.500** ^,^ †	
*M*	26.48	28.30	17.81	38.26	30.07	16.26	97.26
*SD*	7.65	5.89	7.22	9.63	12.83	8.05	14.05

Note

False discovery rate (FDR; Benjamini & Hochberg, 1995) correction of the *p*‐values (implemented using the *p*.adjust function in R) was applied across the six bivariate associations of interest, that is, between mathematics and each individual WM task.

* *p* < .05, one tail

** *p* < .01, one tail

*** *p* < .008, one tail

^†^ *p* < .05, one tail, FDR correction.

### Additional analyses

**Figure 3:**
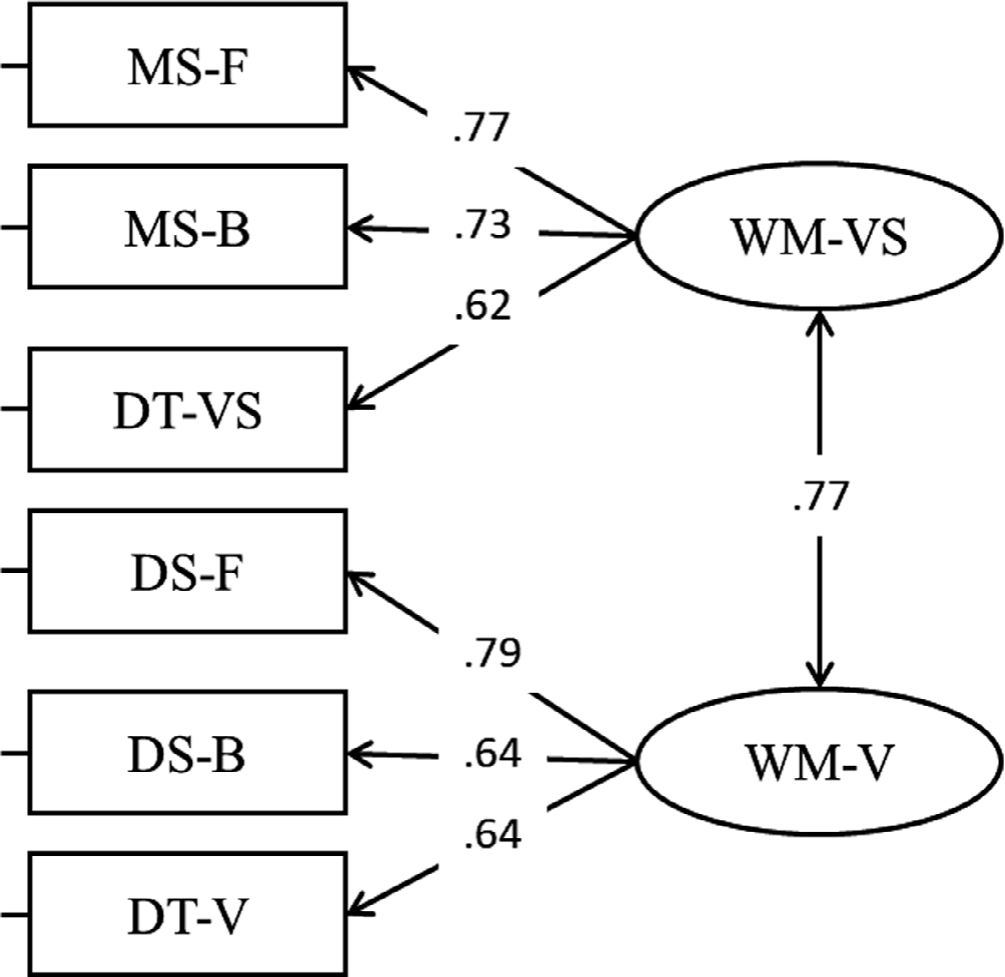
Loadings and correlations for Model 1.

**Figure 4:**
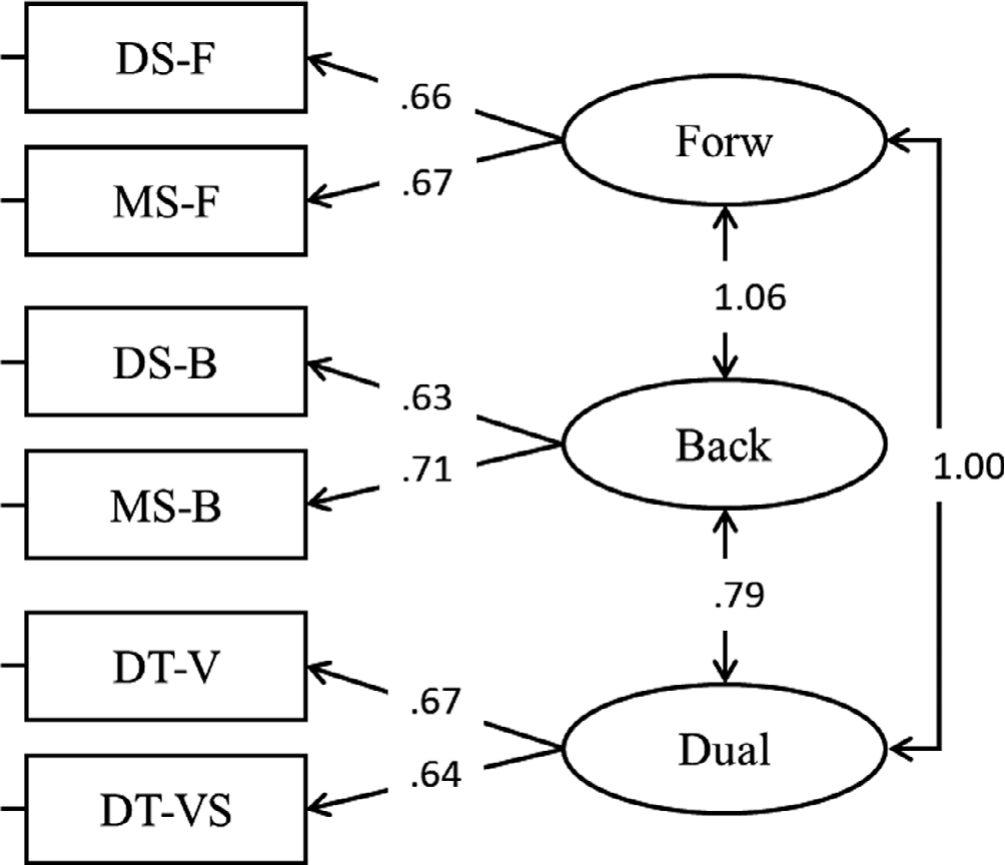
Loadings and correlations for Model 2. Correlations of 1 or higher indicate that factors are not empirically distinguishable.

**Figure 5:**
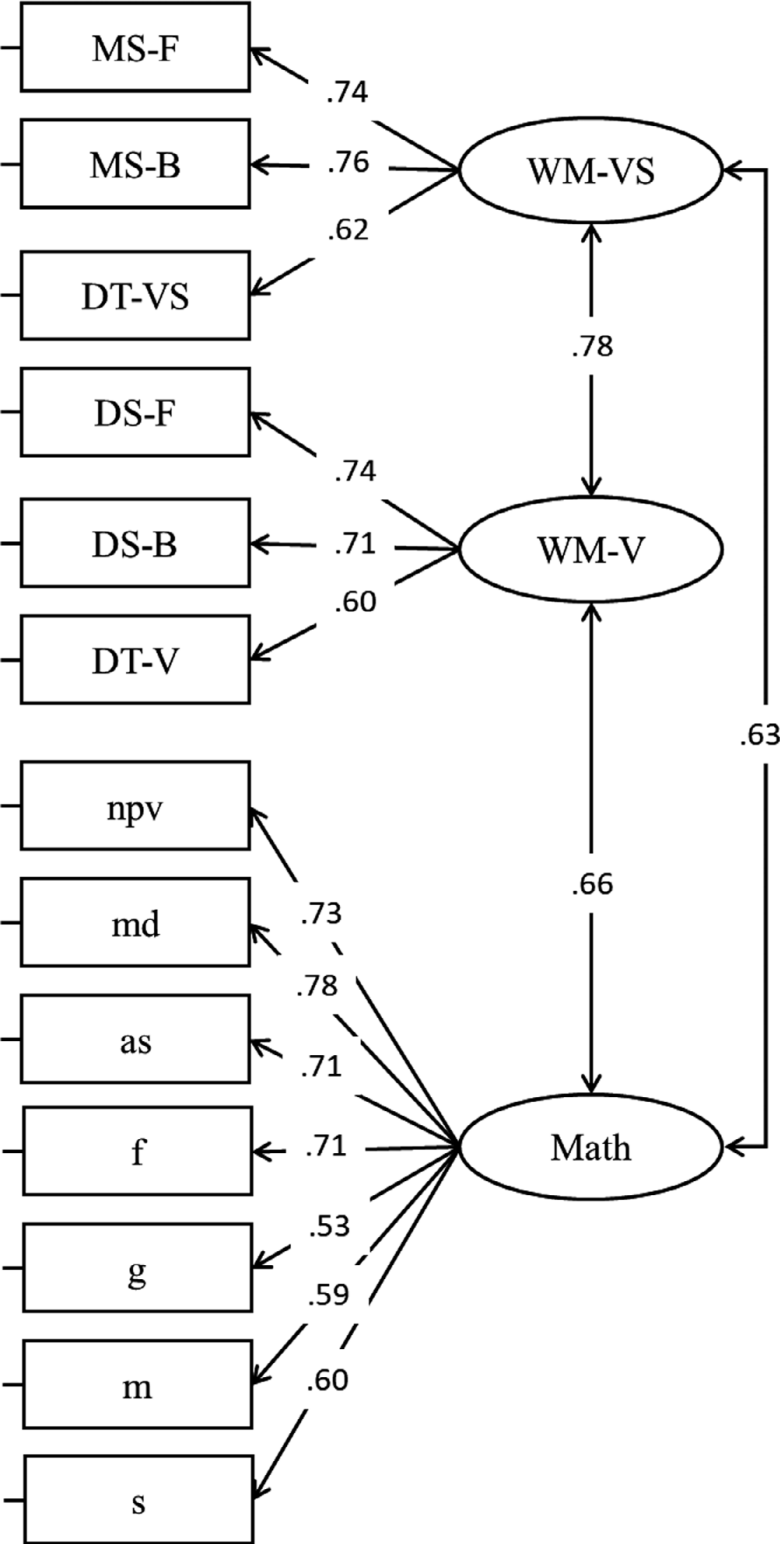
Loadings and correlations for Model 3. Correlations of 1 or higher indicate that factors are not empirically distinguishable.

All the analyses were replicated using a latent modelling approach. In the first step, a CFA was fitted with two factors, verbal working memory and visuospatial working memory. The fit of the model was satisfactory, *χ* ^2^(8) = 9.90, *p* = .272, *RMSEA* = .05, *SRMR* = .04, *CFI* = .99; all loadings were statistically significant as well as the correlation between verbal WM and visuospatial WM (Figure 3). Alternative models were tested, and we fitted a model creating a latent factor including forward span (both verbal and visuospatial tasks), backward span (both verbal and visuospatial tasks), and dual span (both verbal and visuospatial tasks) (Model 2, Figure 4). However, in this model the latent correlation between forward and backward was exceeding one, meaning that these two aspects are very strongly related and should be included in the same factor (Figure 4). For this reason, the distinction between two factors, verbal working memory and visuospatial working memory, was maintained. We therefore decided to go further and test an additional model including a third factor, that is, mathematics. To do so, we first created an individual score for each topic and the resulting scores were used to create a latent variable, that is, mathematics. The fit of the model (Model 3) was adequate, *χ* ^2^(62) = 107.09, *p* < .001, *RMSEA* = .081, *SRMR* = .068, *CFI* = .914 (Model 3; Figure 5). The correlation matrix obtained in Model 3 was used to perform variance partitioning (see Giofrè *et al.*, 2014 for a similar procedure). Results were very similar to those obtained using the observed variables, with visuospatial WM only explaining 7.7% of the variance, while verbal WM was explaining about 15.6% of the total variance, while most of the variance was shared between these two variables, that is, 23.8%. These results, although based on a relatively small sample size, confirm the results obtained above using observed variables, rather than latent factors. The VIF for the model including both verbal WM and visuospatial WM was lower than 2.

The effect of age is moderate and statistically significant when the overall sample is considered (*r*s > .245, *p*s < .01) (see also Logie & Pearson, 1997; Huizinga *et al.*, 2006). Therefore, analyses on the overall sample were repeated controlling for age in months; that is, for each individual WM task, we performed a regression including age as a predictor and each individual WM task as the responding variable, and residuals were then saved and used in subsequent analyses. Results varied very little, significant paths remained significant and changed very little in terms of magnitude. The effect of age was not statistically significant within each year of assessment, and when performing a series of partial correlations controlling for age in months in each year of assessment, results were very similar in terms of magnitude and changed very little. As for the variance partitioning, results were very similar: 22.2% of the variance was shared, 18.5% was explained by verbal WM, and 9.1% was explained by visuospatial WM.

There is a disagreement in the current literature on whether performance on the forward and backward versions of the span (both verbal and visuospatial) is similar or different, with children recalling fewer items in the backward version of the span, which should require more attentional resources (see Donolato, Giofrè, & Mammarella, 2017 for a review). We therefore decided to compare performance in the forward and backward visuospatial and verbal span in the current sample using a series of repeated‐measures ANOVAs. As for the visuospatial span, we found a statistically significant difference between the two versions of the span, *F*(1, 100) = 72.07, *p* < .001, *Cohen’s d* = 0.77, with children recalling more items in the forward version of the span than in the backward. The opposite pattern was found for the verbal span, *F*(1, 100) = 13.41, *p* < .001, *Cohen’s d* = −0.34, with children recalling more items in the backward version of the span, but these differences were somewhat smaller in terms of the effect size compared to the visuospatial WM ones.

**Figure 6:**
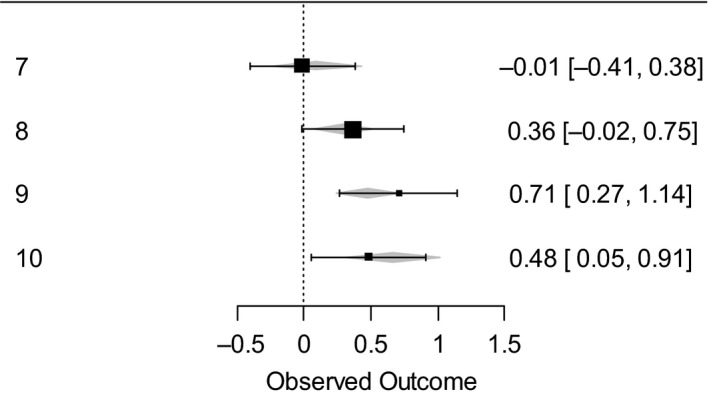
Fisher's *Z* transformed correlations for the relationship between mathematics and dual task spatial as function of age. Error bars represent 95% confidence intervals, while grey diamonds represent predicted effects at each age.

The correlation between Age and Grade was very high (*r* = .94), meaning it is very hard to distinguish between the two. However, it could be argued that the shown pattern of links between WM and mathematics might reflect the test content rather than be evidence of a developmental shift. We originally decided to use grades rather than the actual age of the children in the analysis as this reflects the mathematics they have experienced. To address this issue, however, we performed a series of meta‐analyses dividing the sample into different ages, rather than grades, and comparing the correlations within the age groups, that is, within seven‐year‐olds, eight‐year‐olds, etc. In this analysis, the effect of age as a moderator was investigated. The analytic strategy adopted in this meta‐analysis followed the guidelines proposed by Borenstein *et al. *(2009), and by Schwarzer, Carpenter, and Rücker (2015). R was used in all the analyses (R Core Team, 2018), and meta‐analyses were performed using ‘metafor’ (Viechtbauer, 2010) package. All values were transformed into the Fisher’s *Z* scale before computing the meta‐analysis (see Borenstein *et al.*, 2009 for more details). Estimated coefficients were obtained using the ‘restricted maximum likelihood’ method, which is set by default in the ‘metafor’ package functions. Age did not reach statistical significance as a moderator of the relation between math performance with forward word span, backward word span, verbal dual task, forward matrices, and backward matrices (*p*s > .302). However, as far as the dual task spatial is concerned, we found a statistically significant effect of age, *B* = .016, *p* = .0318 (Figure 6). Showing that pattern of relationship tends to be higher with older children.

## Discussion

This paper aimed to investigate the independent contributions of visuospatial and verbal working memory to mathematical performance in 6‐ to 10‐year‐old children (Years 2–5 in the United Kingdom).

From the correlation analyses (Table 1), we can see that all elements of WM are correlated both with each other and with mathematics, with mathematics being most strongly correlated with backward word span and backward matrices. These correlations were determined after covarying for age, indicating that the relationship with these WM components is relatively stable. These results suggest that there is an element of the task inherent in backward tasks that lends them to being more highly related to mathematics than forward tasks. This is potentially the need for more active processing than is required for forward tasks, which are often viewed as requiring fewer attentional resources (e.g., Passolunghi & Cornoldi, 2008; for a description of tasks whereby active tasks require an additional level of manipulation see Vecchi & Cornoldi, 1999; Vecchi, Richardson & Cavallini, 2005). Further, backward tasks facilitate rehearsal (Conway, Kane, Bunting, Hambrick, Wilhelm & Engle, 2005), with the stimuli being repeated sub‐vocally (for verbal tasks; Baddeley, 1992; Smith, Jonides, Marshuetz & Koeppe, 1998) or in terms of ocular movements (for visuospatial tasks; Tremblay, Saint‐Aubin & Jalbert, 2006) a number of times in order for the participant to accurately reverse the order, producing the final item each time (i.e., *n*, *n* − 1, *n* − 2, etc.) until the entire list has been reversed. This would in itself improve recall if children were afforded the opportunity to rehearse the sequences.

Looking more specifically at the aim of the research, results show that 47.4% of the variance of mathematics performance can be explained by the working memory measures used. Variance partitioning demonstrates that this can be broken down into 15.9% unique variance explained by verbal WM, 7.7% unique variance explained by visuospatial WM, and 23.8% shared variance between verbal WM and visuospatial WM. Unique variance is interpreted as the amount of variance explained by measures of that component of WM, over and above the influence of all other variables measured, for example, that of verbal WM is the variance accounted for by verbal measures over and above the influence of all other measures taken. Here, we see the greatest proportion of unique variance accounted for by verbal measures, followed by visuospatial measures. The largest proportion of variance accounted for by the model is that of shared variance between measures that cannot be attributed solely to verbal or visuospatial measures. This pattern of results is consistent with the findings of Allen *et al*, (2019), but suggests that the influence of verbal‐numeric tasks may not be as great as suggested by Raghubar, Barnes, and Hecht (2010) in their review, beyond the influence of non‐numeric verbal tasks, as non‐numeric verbal tasks also account for a portion of unique variance in mathematics of a similar magnitude Allen *et al*, (2019). Allen *et al*, (2019) used a numeric span, making the findings difficult to generalize to verbal WM as a whole. The magnitude of the influence of WM measures remains stable compared to other studies in the field who identify a similar percentage of variance accounted for (see Giofrè, Donolato & Mammarella, 2018; Kyttaelae & Lehto, 2008for similar results). The amount of shared variance evident in the model may also be related to the previously mentioned strategy choices made by the children (Hecht, 2002; Keeler & Swanson, 2001), for example, visuospatial tasks where children recode the locations as words may draw on both sources of WM. Without recording strategy choice, it is impossible to take this explanation beyond speculation, leaving the potential for future research to investigate whether strategy choice influences the amount of shared variance explained in the models. It is worth noting, however, that this conclusion is very tentative since this study did not differentiate between numeric and non‐numeric verbal tasks, and hence, the percentages of explained variance are compared across studies that use different methods and tasks.

A further aim of the study was to assess whether the WM contributions to mathematics changed with the age of the child as a result of a developmental shift around this time (e.g., De Smedt *et al.*, 2009). We chose to divide the children based on their year group for the analysis because this would be the most appropriate way of controlling for the level of schooling of each child, and thus their exposure to different mathematical concepts. Introducing bias in this is lessened as the year group‐based mathematics tests each contained an equal number of questions relating to the areas outlined by the National Curriculum (number, place, and value [*n* = 4]; multiplication and division [*n* = 4]; addition and subtraction [*n* = 4]; fractions, decimals, and percentages [*n* = 4]; geometry [*n* = 3]; measurement [*n* = 3]; statistics [*n* = 3]) and were designed to test the specific requirements of the National Curriculum for each year group. Hence, two children, both aged seven, but in years 2 and 3, would each receive a mathematics assessment relating to the topics they had been taught to that point. This helps to establish an understanding of learning in relation to teaching, which could not be accurately compared otherwise. Chronological age comparisons would be less appropriate in this situation given the different topics each child has been taught, based on their month of birth. Drawing a cut‐off for age between January and December or September and August is an arbitrary designation, particularly when the difference in age may be of less than a month. Therefore, using the academic calendar in this situation is more appropriate as the children assigned to each year group will have experienced the same level of schooling.

Interestingly, verbal span backwards showed the strongest correlation with mathematics from Year 2 to Year 4; only in Year 5 was this correlation overtaken by visuospatial tasks. This is contrary to our initial prediction and to previous work that has identified a strong influence of visuospatial WM in younger children (e.g., Bull, Espy & Wiebe, 2008; Holmes & Adams, 2006). One possible explanation for this is that all information is presented as words in a written mathematics test, potentially confounded by research showing the presence of reading difficulties relating to difficulties in areas of mathematics (Gersten, Jordan & Flojo, 2005). While we attempted to mediate the influence of reading ability by offering children the opportunity to have questions read aloud, the only way to negate this influence completely would be to present all questions only orally, providing written copies of diagrams where necessary (see Booth & Thomas, 1999 for an example of this method). However, this method of presentation would still draw heavily on verbal WM as children would be required to recall larger amounts of verbal information, for which they only had the opportunity to hear once. As regards formal mathematical testing, written presentation is the preferred method in schools; hence, understanding the influence of WM components when problems are presented in this way will be more beneficial in the long term to the development of interventions, as this will develop an understanding of a child’s ability to work in the manner in which they will be tested.

Following on, considering the later influence of dual tasks on mathematics, as children get older, the type of questions they are asked to complete become more demanding, often containing multiple steps within one question. Inherent in this is the requirement to process larger volumes of information simultaneously for each question, and this requires attentional control resources and higher cognitive processing to a greater extent (see Giofrè, Mammarella, & Cornoldi, 2013 for a similar argument). As such, it follows that a WM task that requires an additional level of manipulation is likely to be more representative of the kinds of processes required for mathematics questions written for older children. Geary, Hoard, Byrd‐Craven, Nugent, and Numtee (2007) identified a number of WM mediators for both simple and complex mathematics questions relating to this idea. Further, visuospatial tasks became more strongly correlated with mathematics from Year 3 onwards. The relationship with backward matrices in Years 3 and 5 fits with the assumption that a more active task aligns more readily with demanding mathematics tasks, which require more than simple repetition to complete (Friso‐van den Bos, van der Ven, Kroesbergen & van Luit, 2013; Giofrè, Mammarella, & Cornoldi, 2013). This finding is consistent with the observation that highly controlled WM processes tend to be more strongly related to higher cognitive abilities both in typically developing children (Cornoldi, Orsini, Cianci, Giofrè, & Pezzuti, 2013) and in particular populations (Cornoldi, Giofrè, Calgaro, & Stupiggia, 2013). Further, questions presented to older children often contain additional information in the form of tables and diagrams, which would serve to engage the visuospatial components of WM more readily than the simpler presentations (see Reuhkala, 2002 for a similar argument).

There are limitations intrinsic to the study design that further research should seek to address, alongside the above suggestion regarding strategy choice. The main difficulty when administering the tests was the selection of dual tasks used with such young children. Children in Year 2 (6–7 years) struggled considerably to comprehend the dual tasks, and as such did not manage to successfully complete the secondary task alongside the primary task in most cases. In future, it would be beneficial to develop a more easily comprehensible dual task that younger children are able to understand sufficiently well as to be able to complete both elements in order to establish an accurate measure of their capabilities in these kinds of tasks. Further, a sample only containing typically developing children is unable to highlight any potential differences between typical and atypical populations. Given the known differences in WM capacity between typical and atypical populations (e.g., Swanson, 1993), it would be informative to collect data demonstrating the longitudinal differences in the contributions of WM to mathematics in these populations to understand whether these are entirely distinct from typical populations or whether they exhibit any overlap. From such work, it would be possible to further understand whether those with mathematical difficulties demonstrate a pattern of developmental delay, or a distinct cognitive profile to typically developing children. We used regressions in order to control for shared variance between variables for the year‐to‐year assessment (Loehlin, & Beaujean, 2017). However, more sophisticated methods are also available (e.g., Gaussian Graphical Model), which allow accessing a conditional dependence/independence of several variables within one model in each group (Costantini *et al.*, 2015; Epskamp & Fried, 2018). In the present report, we decided not to use these methods because of the relatively small sample size, but these methods could successfully be used in future studies with larger samples. Due to the limited sample size within, we decided not to statistically compare correlations coming from independent samples. Future studies with larger sample sizes should be performed to address this issue, for example, using more sophisticated techniques such as Multigroup Confirmatory Factor Analyses or Multigroup Structural Equation Modelling. Finally, future studies should try to compute separated scores for different mathematical subareas, such as, number and geometry. We decided not to perform such analyses here because this would have increased the number of statistical comparisons, and this was not ideal with the current sample size. Given that we used the same WM measures for all ages, but year‐specific maths tasks, it could be argued that the different tasks on a same topic still differed on allocation of visuospatial and verbal WM resources for task completion and caused the reported correlational patterns, for example, Year 2 statistics might have differed from Year 5 statistics and required different cognitive effort in comparison with tasks for other year groups. Future studies should test this hypothesis.

The findings presented above have important implications for educational research as well as for educators in terms of developing interventions to improve mathematical attainment in those with poor mathematical attainment. In order to improve mathematical attainment for those children who demonstrate mathematical difficulties, first a comprehensive understanding of the ways in which WM supports mathematical development is necessary. The results of this study indicate some potential longitudinal changes in the influence of WM components on mathematical attainment, however, also suggest stable elements of influence. Although this paper is only able to identify age‐related differences in the contributions of WM components to mathematical performance at a single point in time in children of different ages, it suggests that future work may seek to identify whether these changes also occur within individuals over development. In a similar vein, we decided to use grade‐specific math assessments in different grades, which is the standard in studies investigating mathematics. However, there is no guarantee on how the math outcomes of one grade are comparable to those of another grade. While the exact amount of unique variance accounted for by verbal and visuospatial WM components at each of the age groups assessed here remains unknown, due to the constraints of sample size, educators would benefit greatly from understanding how these influences change over the primary schools years. In doing so, interventions can be more specifically targeted to provide children with alternative methods that may be better able to support their mathematical development by employing different elements of their WM.

In conclusion, these preliminary results echo those derived from our previous data Allen *et al*, (2019) that verbal WM and visuospatial WM both make unique contributions to mathematical attainment. Further, verbal tasks continue to account for a larger proportion of unique variance, despite the largest proportion being shared variance between both verbal WM and visuospatial WM. Finally, this work demonstrated a change in the strength of the correlations between measures with age, showing that more complex visuospatial tasks become more highly correlated with mathematics as children become older, while verbal task correlations remain relatively stable.

## Compliance with ethical standards

This research is funded by the Economic and Social Research Council. There are no known conflicts of interest, financial, or otherwise, and all data gathered from human participants were done so following obtaining informed consent.

## Conflicts of interest

All authors declare no conflict of interest.

## Author contributions

Katie Allen (Conceptualization; Data curation; Formal analysis; Investigation; Methodology; Writing – original draft); David Giofrè (Conceptualization; Data curation; Formal analysis; Resources; Software; Writing – review & editing); Steve Higgins (Conceptualization; Supervision; Writing – review & editing); John Adams (Conceptualization; Supervision; Writing – review & editing).

## Supporting information


**Figure S1.** Distribution plot for each task in Year 2.
**Figure S2. **Distribution plot for each task in Year 3.
**Figure S3. **Distribution plot for each task in Year 4.
**Figure S4. **Distribution plot for each task in Year 5.
