# Supplementary material for: Working memory predictors of mathematics across the middle primary school years
Source: Br J Educ Psychol. 2020 Jan 30;90(3):848–69. doi: 10.1111/bjep.12339 (PMC7496726; doi:10.1111/bjep.12339)
Supplement: Supplementary file 1 — Figure S1. Distribution plot for each task in Year 2. Figure S2. Distribution plot for each task in Year 3. Figure S3. Distribution plot for each task in Year 4. Figure S4. Distribution plot for each task in Year 5. [file BJEP-90-848-s001.docx]

Supplementary material


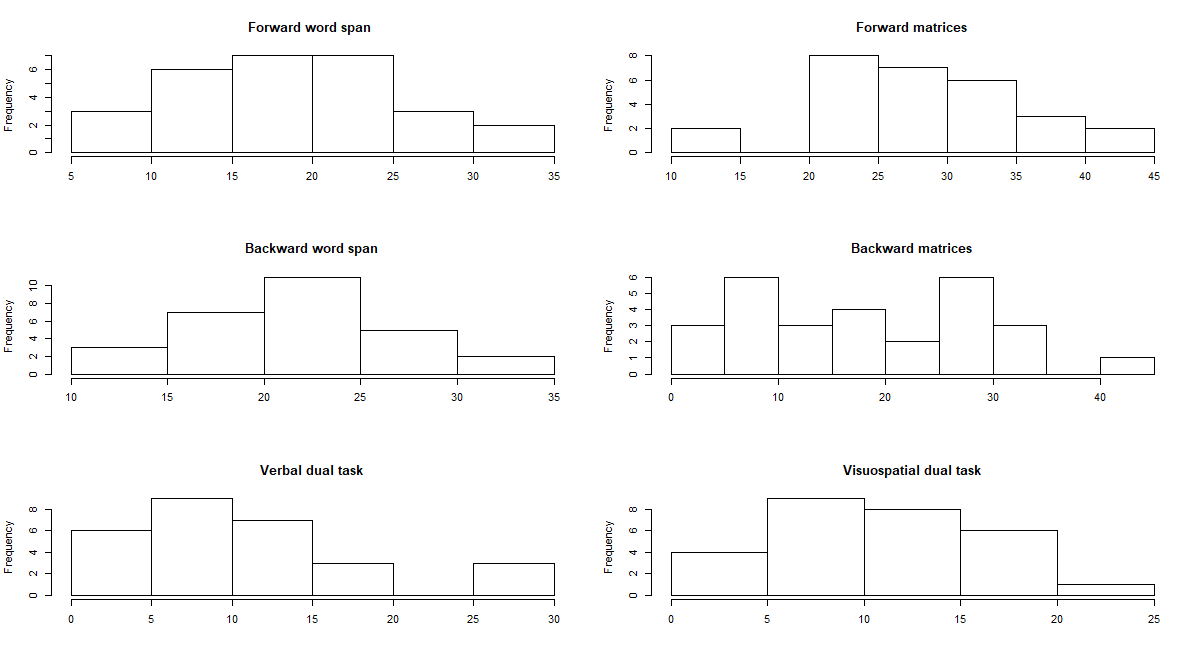


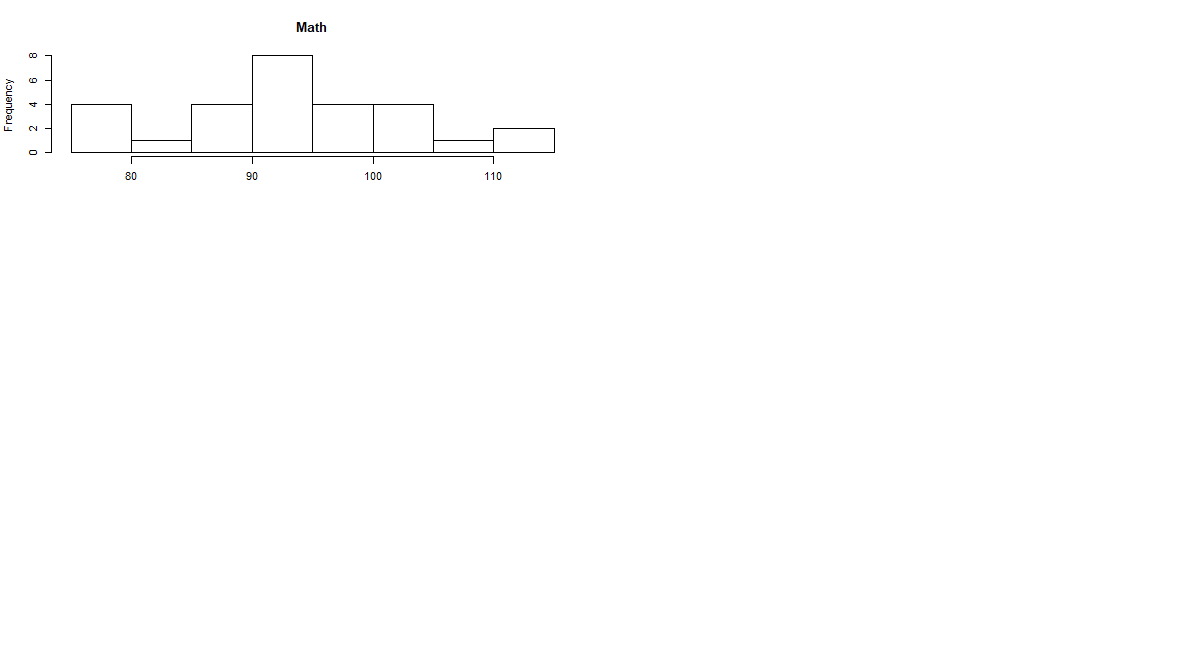


*Figure 1S.* Distribution plot for each task in Year 2.


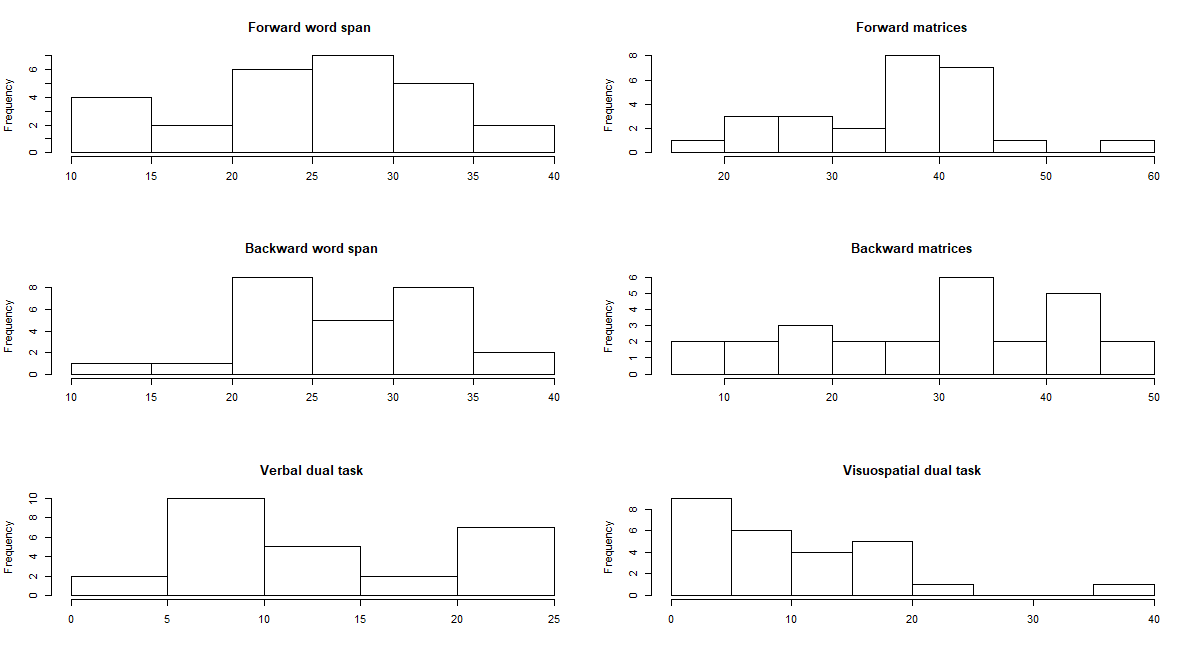

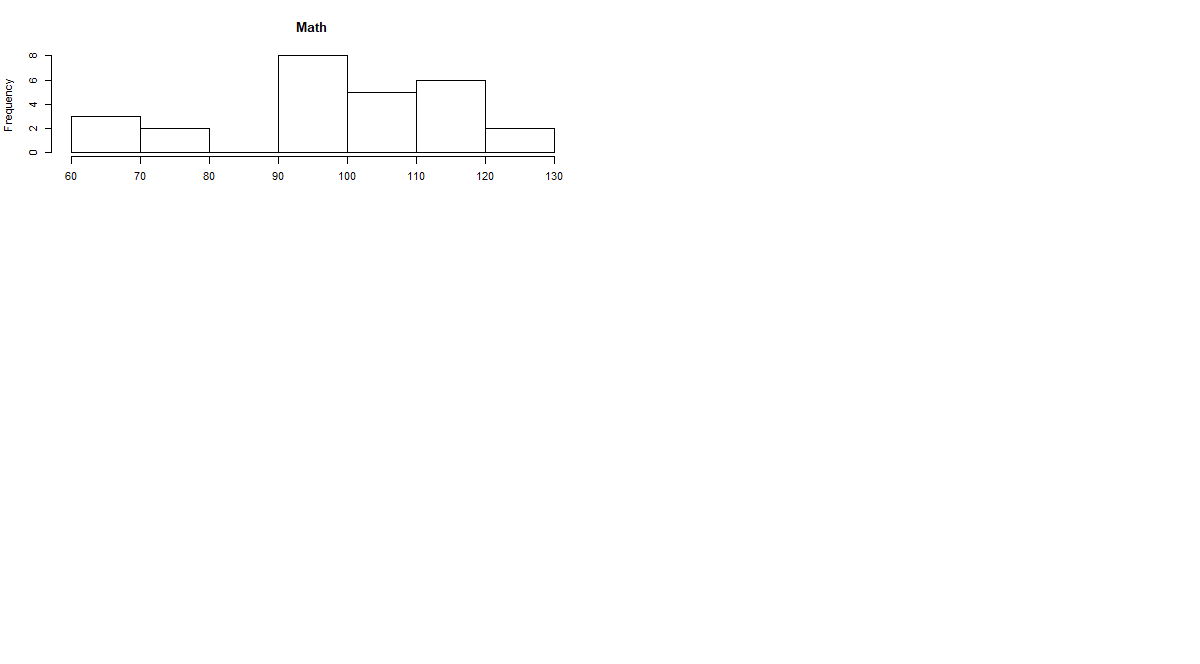


*Figure 2S.* Distribution plot for each task in Year 3.


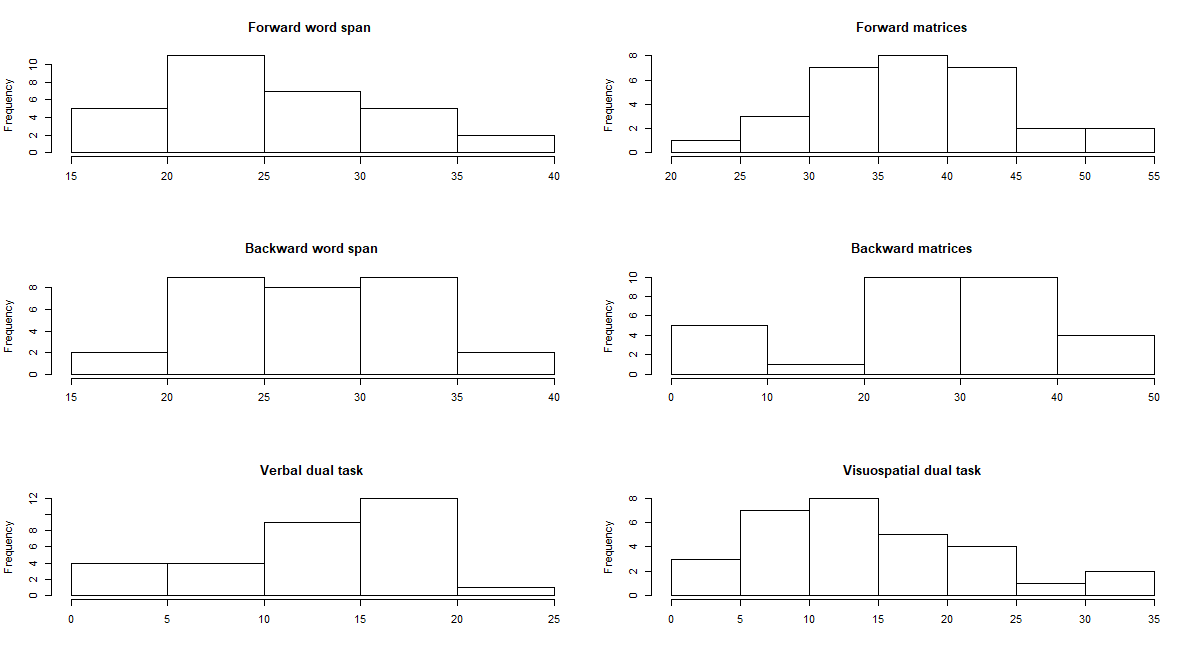

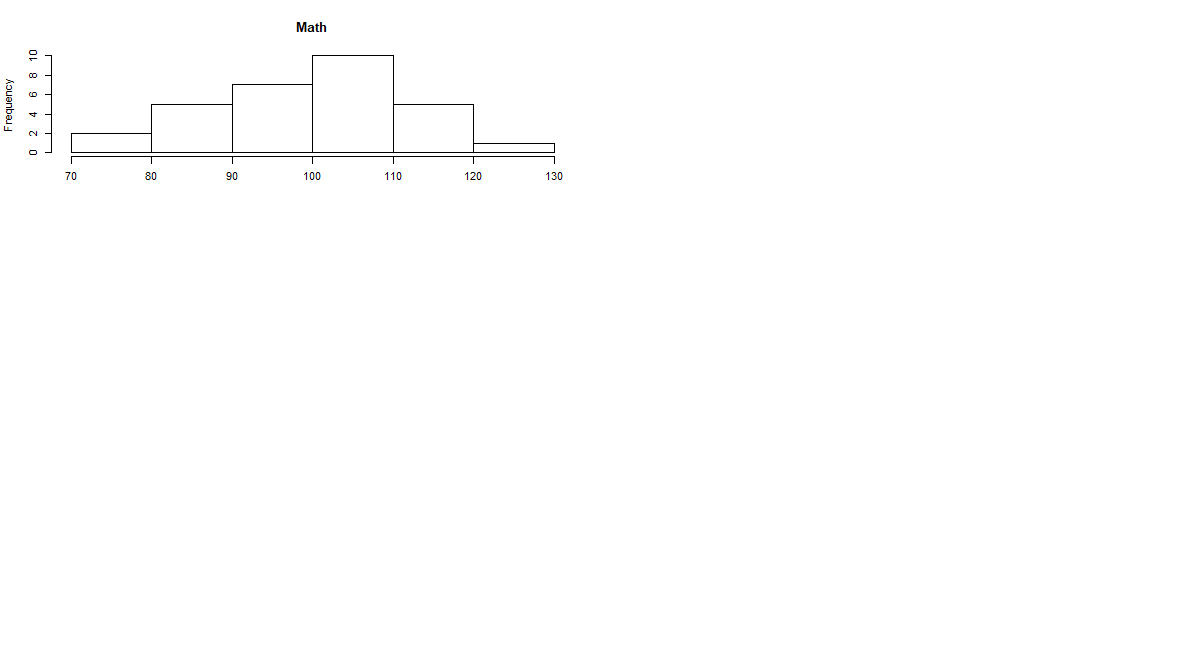


*Figure 3S.* Distribution plot for each task in Year 4.


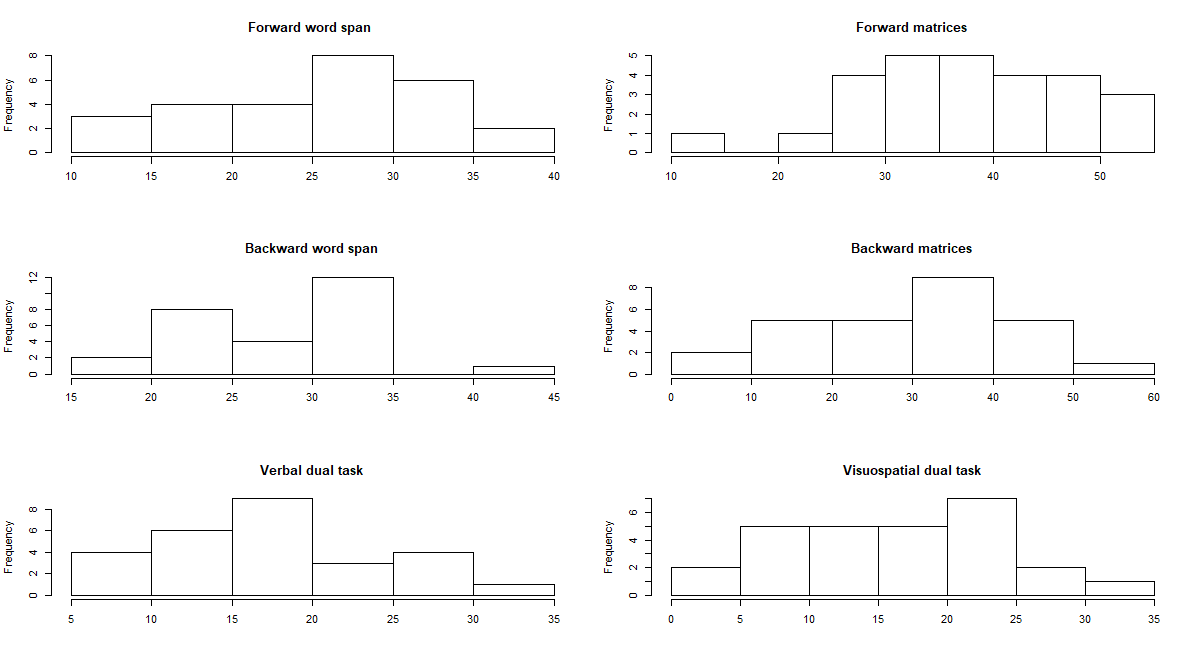

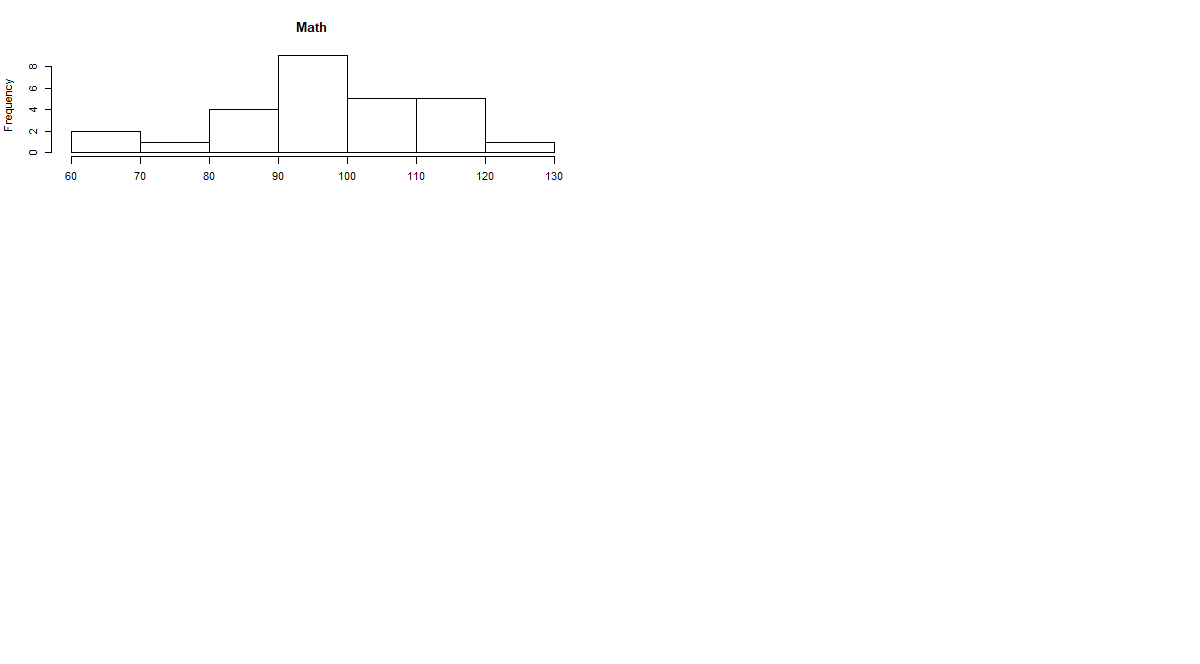


*Figure 4S.* Distribution plot for each task in Year 5.
